# Trends in psychiatric occupational therapy in Japan: A nationwide analysis using the National Database of Health Insurance Claims and Specific Health Checkups of Japan from 2014 to 2022

**DOI:** 10.1002/pcn5.70224

**Published:** 2025-10-27

**Authors:** Tomoyuki Okazaki, Satoshi Asaoka, Chie Kitano, Hitoshi Okamura

**Affiliations:** ^1^ Medical Corporation Kouseikai, Cocoro Hospital Kusatsu Hiroshima Japan; ^2^ Department of Psychosocial Rehabilitation, Graduate School of Biomedical and Health Sciences Hiroshima University Hiroshima Japan

**Keywords:** COVID‐19, health insurance claims data, mental health services, National Database of Health Insurance Claims and Specific Health Checkups of Japan (NDB), psychiatric occupational therapy

## Abstract

**Aim:**

Mental health issues are a significant global concern, with psychiatric occupational therapy (OT) playing a crucial role in non‐pharmacological treatment. In Japan, psychiatric OT is reimbursed under the national medical fee system. This study aimed to elucidate changes in psychiatric OT claims in Japan and analyze these changes by gender and age group.

**Methods:**

The National Database of Health Insurance Claims and Specific Health Checkups of Japan (NDB) Open Data from fiscal years 2014–2022 was used. The number of psychiatric OT claims was analyzed by fiscal year, age group, sex, and region. Simple regression analysis, Mann–Whitney *U* test, and Spearman's rank correlation were used for statistical analysis.

**Results:**

Inpatient psychiatric OT claims significantly increased from 2014 to 2022, particularly among the elderly (≥70 years) and those aged < 20 years. Outpatient claims significantly decreased after the onset of the COVID‐19 pandemic. Females had significantly higher inpatient claims than males, but no significant difference was observed for outpatients. Substantial regional disparities were found in the implementation of psychiatric OT.

**Conclusion:**

The increase in inpatient psychiatric OT claims, especially among the elderly and younger populations, highlights the growing importance of age‐specific interventions. The decline in outpatient claims raises concerns about the impact of the pandemic on community‐based rehabilitation. Policymakers should consider revising reimbursement schemes to support sustained outpatient OT provision and ensure its integration as a core component of inclusive mental health care systems.

## INTRODUCTION

Mental health issues represent a significant social concern, with one in eight individuals globally, or 970 million people, experiencing a mental disorder, according to the World Health Organization (WHO).^1^ In Japan, the estimated number of individuals with mental illnesses is 6.03 million, of whom 266,000 are hospitalized.^2^ Japan is noted for having one of the highest numbers of psychiatric beds among OECD countries, and the average length of stay is also long.^3^ Consequently, a vision has been articulated to transition from hospital‐centered to community‐centered care.

Occupational therapy (OT) plays a crucial role in the non‐pharmacological treatment of individuals with mental illnesses. OT promotes health and well‐being by supporting participation in meaningful occupations that people want, need, or are expected to do.^4^ In mental health settings, various interventions delivered by occupational therapists have been documented, including craft activities, interpersonal communication, psychoeducation,^5^ cognitive function interventions,^6^ exercise programs,^7^ and programs promoting improvements in psychiatric symptoms,^8^ functional independence,^9^ and community participation.^10^ In Japan, psychiatric OT is reimbursed under the national medical fee system and plays a central role in psychiatric rehabilitation for both inpatients and outpatients across diverse age groups and diagnoses. Standard delivery involves group‐based interventions for up to 25 participants for approximately 2 h daily.

In recent years, the COVID‐19 pandemic has significantly affected psychiatric OT services, particularly because of the nature of group‐based interventions. Due to infection control measures, interventions involving close interaction—such as group discussions, cooking, or shared meals—were often restricted. Some hospitals suspended OT services temporarily due to outbreaks or restricted access for outpatients. Conversely, the 2018 revision of the national medical fee schedule allowed psychiatric OT to be counted toward the required functional recovery training in dementia treatment wards, facilitating claims and possibly contributing to the observed increase in older adults.^11^

In recent years, the status of psychiatric OT in Japan has been changing. However, quantitative analyses examining changes in the operational aspects, such as total medical fees, of psychiatric OT in Japan are lacking. Psychiatric OT encompasses a wide range of disorders and age groups. OT defines occupation as all purposeful and valuable life activities for individuals.^12^ It is beneficial to elucidate the characteristics of patients targeted by psychiatric OT in Japan, particularly in relation to the occupations utilized and tasks assigned to participants. A previous study based on a questionnaire survey conducted by the Japanese Occupational Therapist Association (JAOT) reported the characteristics of psychiatric OT participants.^13^ However, this report does not include all medical institutions. Similarly, there is a report based on the number of psychiatric OT claims,^14^ but it merely compares data from single months across different fiscal years and does not examine long‐term trends. To date, no study has quantitatively assessed the potential differences in treatment practices across sex and generations in Japan.

The Japanese Ministry of Health, Labour, and Welfare (MHLW) has published annual data for each treatment provided “by prefecture,” “by month,” and “by sex and age” in the National Database of Health Insurance Claims and Specific Health Checkups of Japan (NDB) as NDB Open Data. Japan, with its universal health insurance system, aggregates domestic receipt information in the NDB Open Data, which is highly comprehensive. Analyzing NDB Open Data will facilitate understanding of medical care trends and the general landscape in Japan. Some studies utilizing NDB Open Data have been published.^15^, ^16^, ^17^, ^18^ Therefore, we considered it beneficial to analyze the actual status of psychiatric OT in Japan. Consequently, we conducted a survey to quantitatively examine the implementation and trends of psychiatric OT by fiscal year, sex, and age group using NDB Open Data. The objectives of this study were (1) to elucidate changes in the number of psychiatric OT claims in Japan and (2) to elucidate changes in these claims by gender and age group. This study aimed to assess the actual situation and needs of psychiatric OT in Japan.

## MATERIALS AND METHODS

### Data collection

The NDB Open Data^19^ comprises data on health insurance claims receipts in Japan, rendering it suitable for analyzing the actual conditions and trends in medical care within the country. The NDB Open Data are based solely on electronically submitted claims and do not include paper‐based claims. This study used data from FY to 2014–2022. Based on data from medical inpatient/outpatient receipts and Diagnosis Procedure Combination (DPC) receipts, the number of claims for each medical practice corresponding to each medical fee point was aggregated by 47 prefectures in Japan, sex, 5‐year age groups, and month. In this study, “psychiatric occupational therapy” was employed from the medical fee items (Table 1).

**Table 1:** Medical practice code numbers used in this study.

Medical practice	Code number	Calculation requirements
Psychiatric occupational therapy	180007410	Psychiatric occupational therapy is aimed at the recovery of the social functioning of people with mental illness. The standard duration is 2 h per patient per day. The number of patients handled by one occupational therapist per day is generally set at 25 per unit. The standard number of patients handled by one occupational therapist per day is 50 or less, with a maximum of 2 units.

The 630 Survey (June 30th survey: national information on mental health and welfare)^20^ is conducted annually on June 30 to comprehend the state of mental health and welfare in Japan, with the results being made publicly available. The survey data provided a detailed breakdown of the number of psychiatric hospital patients by age group, disease categories, and prefectures. It aims to assess the actual situation of patients utilizing psychiatric hospitals, psychiatric clinics, and home nursing stations and is employed in policy measures. The response rate exceeded 95%, and the survey had exhaustive coverage.

### Data analysis

In this study, the term “psychiatric occupational therapy” is specifically defined as services billed under the designated reimbursement code for psychiatric OT within the national fee schedule. Psychiatric OT services rendered in psychiatric settings but not billed under this code, such as those included in bundled payment systems like *child/adolescent psychiatric inpatient care fee* were not captured in the NDB dataset and were therefore excluded from the analysis.

#### Changes by fiscal year

Using NDB Open Data, we analyzed the total number of claims for each fiscal year from 2014 to 2022, encompassing both inpatient and outpatient cases. For inpatient data, the number of claims was treated as a continuous variable, and annual trends were determined. Consequently, a simple regression analysis was conducted, with the fiscal year serving as the explanatory variable and the number of claims as the dependent variable. Conversely, outpatient data exhibited a marked decline in the number of claims beginning in the 2020 fiscal year. To assess the impact of the COVID‐19 pandemic, the data were segmented into two periods: pre‐pandemic (fiscal years 2014–2019) and post‐pandemic (fiscal years 2020–2022). Mann–Whitney *U* test was used to compare the average number of claims between these periods. Additionally, we calculated the percentage change in the number of claims for the fiscal year 2022 relative to the fiscal year 2014 for both inpatient and outpatient data.

#### Changes by age group for each fiscal year

We conducted an analysis using the total number of claims categorized by sex and age groups from the NDB Open Data. Notably, items with ten or fewer aggregated values are masked and represented by hyphens. Furthermore, adhering to the principle of the smallest aggregation unit, one item was masked between inpatient data for fiscal year 2016 and both inpatient and outpatient data for fiscal year 2017. As the masked values were deduced from the totals, all items were masked. Therefore, in this study, we excluded these items and employed the aggregated values for each sex and age group (in 5‐year intervals) of inpatient and outpatient data spanning fiscal years 2014–2022. Additionally, we used data on the number of inpatients by age group from the 630 Survey to discern trends in psychiatric hospitalizations from 2014 to 2022. Based on the age‐specific totals from the NDB public data, the age categories utilized in the 630 Survey (under 20, 20–39, 40–65, 65–74, 75, and over) were standardized. The number of claims per age group for each fiscal year and claims per population were calculated using the population estimates provided by the Ministry of Internal Affairs and Communications.^21^ Additionally, to assess the intensity of intervention, Inpatient claims per Inpatient (as of June 30) was calculated. This metric was determined by using the number of inpatients as of June 30 (from the “630 Survey”) as a proxy for the denominator, as annual total inpatient data is unavailable. This metric served as an indicator of the concentration of psychiatric OT provision relative to the inpatient population size. To analyze the trends in changes from FY2014 to FY2022 for each age group, we performed a simple regression analysis with the fiscal year as the explanatory variable and the number of assessments as the dependent variable. Moreover, to capture the trend in the number of inpatients deemed eligible for psychiatric OT during this period, we used age‐specific data from the 630 Surveys conducted from 2014 to 2022 and calculated the rates of increase or decrease for each age group.

#### Sex difference

The analysis utilized the “by sex and age” totals for fiscal years 2014–2022. We computed the total number of psychiatric OT claims. Mann–Whitney *U* test was conducted to compare the number of claims for each fiscal year, excluding 2016 and 2017 for inpatients, and excluding 2017 for outpatients, categorized by sex.

#### Regional difference

In the analysis of regional disparities, “region” was defined as the geographical location of each medical institution. For the fiscal year 2022, the annual number of inpatient and outpatient claims by prefecture was used. The number of claims per 1000 people was calculated using population estimates from the Ministry of Internal Affairs and Communications, also by prefecture.^21^ Furthermore, inpatient claims per inpatient (as of June 30) were determined using the number of inpatients as of June 30, as reported in the “630 Survey,” as the denominator. The maximum and minimum values for both population‐based and inpatient‐based claims rates were identified. Spearman's rank correlation coefficients were employed to investigate the relationships between (1) the number of beds per 100,000 population^22^ and inpatient/outpatient claims per 1000 population; (2) the number of beds per 100,000 population and inpatient claims per inpatient (as of June 30); and (3) population‐based claim rates and inpatient‐based claim rates.

### Statistical analysis

All statistical analyses were conducted using IBM spss Statistics 29.0 (IBM Corp., Armonk, NY, USA) and HAD.^23^ *p*‐Values were calculated using a two‐tailed approach, with values less than 0.05 being deemed statistically significant.

### Ethical considerations

This study used publicly available data and did not involve individual patient information. Consequently, there were no ethical concerns.

## RESULTS

### Changes by fiscal year

The annual number of psychiatric OT claims is shown in Table 2. The annual number of claims for inpatients exhibited an upward trend, increasing by 16.1% from FY2014 to FY2022. The results of the simple regression analysis, with the year as the explanatory variable and the number of claims as the dependent variable, indicated that the regression coefficient for the year was statistically significant (*p* < 0.01, *R* ^2^ = 0.81).

**Table 2:** Number of psychiatric occupational therapy calculations for each fiscal year (times).

Fiscal year	Inpatient	Outpatient	Notes
2014	20,382,913	439,356	
2015	20,842,918	450,328	
2016	21,337,457	448,448	
2017	21,692,094	456,558	
2018	23,205,699	458,133	
2019	23,885,796	433,857	
2020	25,274,054	260,997	During COVID‐19^a^
2021	25,195,983	255,473	During COVID‐19^a^
2022	23,661,307	253,127	During COVID‐19^a^

^a^ During COVID‐19: Many psychiatric occupational therapy services were temporarily suspended or operated with reduced capacity due to infection control measures, depending on facility‐level policies.

Conversely, the annual number of claims for outpatients demonstrated a downward trend, decreasing by 42.4% from FY2014 to FY2022. Notably, the number of claims declined by 39.8% from FY2019 to FY2020 during the onset of the COVID‐19 pandemic. This decline may reflect a combination of pandemic‐related factors, including infection control measures, temporary service suspension, and staffing constraints in some facilities during the pandemic. Mann‐Whitney U test results revealed a significant difference in the number of claims for outpatients before (447,780.0 ± 9549.8) and after (256,352.3 ± 4040.5) the COVID‐19 pandemic (*p* = 0.02).

### Differences between age groups (inpatients)

**Figure 1:**
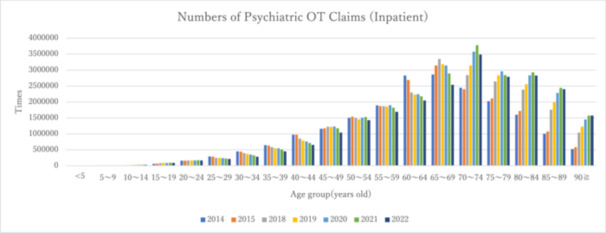
Number of claims for inpatients aged 5 and older by age group from FY2014 to FY2022. OT, occupational therapy.

**Figure 2:**
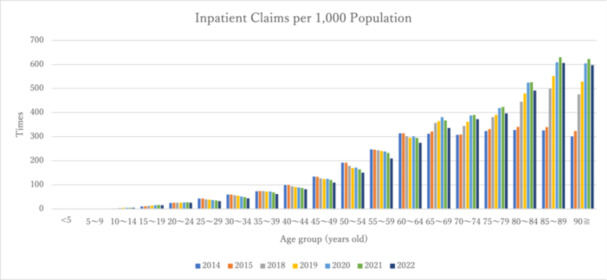
Number of inpatient claims per 1000 population aged 5 and older by age group from FY2014 to FY2022.

Figure 1 illustrates the number of claims for inpatient individuals categorized by 5‐year age groups, whereas Figure 2 depicts the number of claims per population. From FY2014 to FY2022, the data revealed that both the number of inpatient claims and the number of claims per population exhibited an upward trend among the elderly population (approximately 70 years and older), demonstrating the highest values across all age groups. Table 3 presents the trends and rates of change in the number of claims, number of claims per population, and number of claims per inpatient as of June 30, spanning the fiscal years 2014–2022. Table 4 provides data on the number of inpatients categorized by age group, as reported in the 630 Survey.

**Table 3:** Inpatient psychiatric occupational therapy (OT) claims by age group: number of claims, claims per 1000 population, claims per inpatient (as of June 30), and percent change from FY2014 to FY2022.

Fiscal year	<20 years			20–39 years old			40–64 years old			65–74 years old			>75 years old		
	Number of claims	Claims per 1000 population	Claims per inpatient (as of June 30)	Number of claims	Claims per 1000 population	Claims per inpatient (as of June 30)	Number of claims	Claims per 1000 population	Claims per inpatient (as of June 30)	Number of claims	Claims per 1000 population	Claims per inpatient (as of June 30)	Number of claims	Claims per 1000 population	Claims per inpatient (as of June 30)
2014	73,989	3.33	35.03	1,532,583	52.82	71.44	8,339,933	194.75	76.79	5,299,127	310.20	73.37	5,137,281	322.77	59.72
2015	81,550	3.71	37.95	1,515,192	53.28	74.17	8,239,205	192.55	79.57	5,539,861	315.75	76.65	5,467,108	334.97	63.27
2016	No data	No data	No data	No data	No data	No data	No data	No data	No data	No data	No data	No data	No data	No data	No data
2017	No data	No data	No data	No data	No data	No data	No data	No data	No data	No data	No data	No data	No data	No data	No data
2018	99,339	4.66	41.79	1,379,819	50.76	74.59	7,729,025	182.45	83.38	6,180,567	351.11	86.28	7,816,925	434.83	82.01
2019	108,511	5.16	43.61	1,322,719	49.12	75.42	7,539,319	178.15	85.84	6,316,785	363.16	91.66	8,598,462	465.03	90.21
2020	114,728	5.53	49.60	1,296,646	48.17	81.88	7,634,063	179.78	90.88	6,714,479	385.34	97.32	9,514,130	511.46	96.76
2021	124,249	6.10	47.24	1,223,878	46.09	80.96	7,401,476	174.68	91.67	6,669,733	380.24	99.06	9,776,633	523.60	100.60
2022	116,869	5.84	41.98	1,103,702	41.91	75.14	6,840,093	161.46	87.64	6,019,183	356.76	94.40	9,581,443	494.81	96.17
Percent change from FY2014 to FY2022	58.0%	75.5%	19.8%	−28.0%	−20.6%	5.2%	−18.0%	−17.1%	14.1%	13.6%	15.0%	28.7%	86.5%	53.3%	61.0%

**Table 4:** Number of inpatients by age group for each fiscal year (630 Survey).

Fiscal year	<20 years	20–39 years old	40–64 years old	65–74 years old	>75 years old	Unknown
2014	2112	21,454	108,600	72,222	86,018	
2015	2149	20,428	103,553	72,273	86,403	
2016	2355	19,968	100,777	73,523	89,783	
2017	2387	19,382	97,212	72,539	92,406	246
2018	2377	18,498	92,691	71,633	95,319	297
2019	2488	17,539	87,832	68,915	95,318	4
2020	2313	15,836	84,005	68,993	98,326	3
2021	2630	15,117	80,744	67,330	97,182	4
2022	2784	14,689	78,051	63,760	99,632	4

For each age group, a single regression analysis was performed, utilizing the number of claims, claims per population, and number of claims per inpatient (as of June 30) as dependent variables, with the fiscal year as the explanatory variable (Table 5).

**Table 5:** Trends in the number of psychiatric occupational therapy (OT) claims by age group (inpatients).

Age group		*β*	SE	Adjusted *R* ^2^	*p*	VIF
<20 years old	Number of claims	0.97	631.75	0.94	0.00**	1.00
	Inpatient claims per 1000 population	0.99	0.03	0.96	0.00**	1.00
	Annual claims per inpatient (as of June 30)	0.79	0.46	0.55	0.04*	1.00
20–39 years old	Number of claims	−0.98	4740.22	0.95	0.00**	1.00
	Inpatient claims per 1000 population	−0.93	0.22	0.83	0.00**	1.00
	Annual claims per inpatient (as of June 30)	0.67	0.42	0.34	0.10	1.00
40–64 years old	Number of claims	−0.96	22,562.77	0.90	0.00**	1.00
	Inpatient claims per 1000 population	−0.95	0.54	0.88	0.00**	1.00
	Annual claims per inpatient (as of June 30)	0.92	0.33	0.82	0.00**	1.00
65–74 years old	Number of claims	0.80	47,803.69	0.57	0.03*	1.00
	Inpatient claims per 1000 population	0.87	2.12	0.72	0.01*	1.00
	Annual claims per inpatient (as of June 30)	0.95	0.45	0.89	0.00**	1.00
>75 years old	Number of claims	0.98	59,409.22	0.95	0.00**	1.00
	Inpatient claims per 1000 population	0.96	3.37	0.91	0.00**	1.00
	Annual claims per inpatient (as of June 30)	0.97	0.58	0.93	0.00**	1.00

*Note*: P, simple linear regression. Abbreviation: VIF, variance inflation Factor.

* *p* < 0.05

** *p* < 0.01.

Significant increases in the number of claims and claims per population were observed in the age groups < 20, 65–74, and ≥75 years. Conversely, significant decreases were noted in the 20–39 and 40–64 age groups. Additionally, the number of claims per inpatient (as of June 30) exhibited significant increases across all age groups, except for the 20–39 age group.

### Differences between age groups (outpatient)

**Figure 3:**
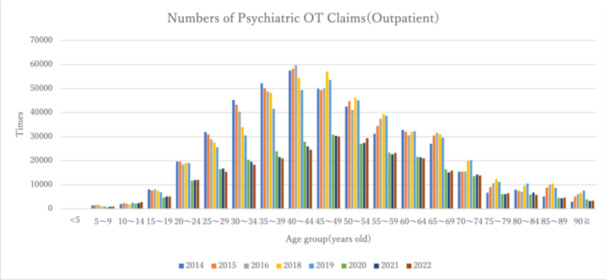
Number of claims for outpatients aged 5 and older by age group from FY2014 to FY2022. OT, occupational therapy.

**Figure 4:**
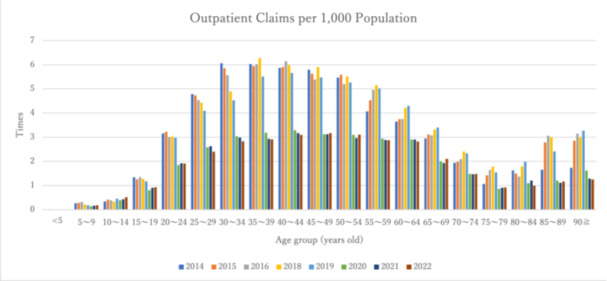
Number of outpatient claims per 1000 population aged 5 and older by age group from FY2014 to FY2022.

Figures 3 and 4 and Table 6 show the number of outpatient claims and claims per 1000 population by age group based on data from fiscal years 2014 to FY2022. The number of claims remained consistently high, particularly among individuals in their 40s. Claims per 1000 population were high among those in their 30s to 50s.

**Table 6:** Outpatient psychiatric occupational therapy (OT) claims by age group: number of claims, claims per 1000 population, and percent change (FY2019–FY2020).

Fiscal year	<20 years		20–39 years old		40–64 years old		65–74 years old		>75 years old	
	Number of claims	Outpatient claims per 1000 population	Number of claims	Outpatient claims per 1000 population	Number of claims	Outpatient claims per 1000 population	Number of claims	Outpatient claims per 1000 population	Number of claims	Outpatient claims per 1000 population
2014	11,513	0.52	148,979	5.13	213,810	4.99	42,380	2.48	22,674	1.42
2015	11,535	0.52	143,813	5.06	218,683	5.11	45,874	2.61	30,423	1.86
2016	12,004	0.55	136,654	4.90	218,766	5.13	47,143	2.67	33,881	2.00
2017	No data	No data	No data	No data	No data	No data	No data	No data	No data	No data
2018	10,460	0.49	128,806	4.74	229,026	5.41	50,840	2.89	39,001	2.17
2019	10,316	0.49	116,730	4.33	218,967	5.17	49,820	2.86	38,024	2.06
2020	7475	0.36	72,446	2.69	130,692	3.08	30,023	1.72	20,361	1.09
2021	8208	0.40	69,854	2.63	127,634	3.01	29,376	1.67	20,394	1.09
2022	8779	0.44	66,517	2.53	128,083	3.02	29,659	1.76	20,089	1.04
Percent change from FY2019 to FY2020	−27.5%	−26.5%	−37.9%	−37.9%	−40.3%	−40.5%	−39.7%	−39.8%	−46.5%	−46.8%

Comparing the period before the COVID‐19 pandemic (FY2019) with the period after its onset (FY2020), both the number of claims for psychiatric OT and the number of claims per 1000 population decreased in all age groups.

### Sex difference

The number of psychiatric OT claims for inpatients and outpatients is shown in Table 7. The results of the Mann–Whitney *U* test showed that the number of claims for inpatients was higher for females (mean ± SD = 12,695,127.3 ± 1,365,504.9) than for males (10,511,816.3 ± 596,198.8; *p* = 0.02, *r* = 0.67), and there was no significant difference in the number of claims for outpatients by sex (males: mean ± SD = 175,841 ± 49,476.1; females: 199,122.3 ± 49,034.2; *p* = 0.08, *r* = 0.45).

**Table 7:** Number of psychiatric occupational therapy (OT) claims by sex for each fiscal year.

Fiscal year	Sex	Inpatient	Outpatient	Notes
2014	Male	9,695,731	210,787	
	Female	10,687,182	228,569	
2015	Male	9,863,607	215,990	
	Female	10,979,309	234,338	
2016	Male	N/A	214,743	No inpatient data
	Female	N/A	233,705	No inpatient data
2017	Male	N/A	N/A	No data
	Female	N/A	N/A	No data
2018	Male	10,554,923	214,612	
	Female	12,650,752	243,521	
2019	Male	10,748,530	201,387	
	Female	13,137,266	232,470	
2020	Male	11,268,788	120,572	
	Female	14,005,258	140,425	
2021	Male	11,128,228	116,429	
	Female	14,067,741	139,037	
2022	Male	10,322,907	112,214	
	Female	13,338,383	140,913	

### Regional difference

Tables 8.1 and 8.2 present the billing status of psychiatric OT in each prefecture. In the analysis of inpatient claims per 1000 population, Saga Prefecture demonstrated the highest incidence at 648.3, whereas Kyoto Prefecture exhibited the lowest at 69.6. In terms of outpatient claims per 1000 population, Nagano Prefecture recorded the highest rate at 10.0, contrasting with Oita Prefecture, which reported the lowest at 0. Saga Prefecture also showed the highest inpatient claim rate per patient at 144.4, whereas Kyoto Prefecture had the lowest at 41.3. A significant positive correlation was observed between the number of beds per 100,000 population and both inpatient claims per 1000 population (*r* = 0.92, *p* < 0.01) and outpatient claims per 1000 population (*r* = 0.31, *p* = 0.03). Moreover, significant positive correlations were identified between the number of beds per 100,000 population and inpatient claims per inpatient (as of June 30) (*r* = 0.32, *p* = 0.03), and between inpatient claims per 1000 population and inpatient claims per inpatient (as of June 30) (*r* = 0.63, *p* < 0.01).

**Table 8.1:** Number of claims, claims per 1000 population, and inpatient claims per inpatient (as of June 30) by prefecture, FY2022 (Hokkaido‐Mie).

Prefecture	Number of claims (inpatient)	Number of claims (outpatient)	Inpatient claims per 1000 population	Outpatient claims per 1000 population	Inpatient claims per inpatient (as of June 30)
Hokkaido	1,339,106	8509	260.53	1.66	88.46
Aomori	308,091	4613	255.89	3.83	88.35
Iwate	277,796	5540	235.22	4.69	89.38
Miyagi	394,846	2499	173.18	1.10	93.08
Akita	236,611	4249	254.42	4.57	71.90
Yamagata	283,003	3125	271.86	3.00	94.11
Fukushima	341,456	4431	190.76	2.48	81.34
Ibaraki	399,975	1498	140.84	0.53	72.54
Tochigi	326,508	9317	171.04	4.88	86.22
Gunma	448,715	1102	234.56	0.58	100.03
Saitama	1,115,327	11,810	152.01	1.61	121.10
Chiba	740,944	3918	118.25	0.63	77.68
Tokyo	1,112,121	22,108	79.22	1.57	66.08
Kanagawa	955,161	7298	103.46	0.79	85.50
Niigata	484,621	10,071	225.09	4.68	96.17
Toyama	184,359	3125	181.28	3.07	67.21
Ishikawa	246,250	7620	220.26	6.82	80.68
Fukui	165,933	3014	220.36	4.00	94.17
Yamanashi	143,295	1072	178.67	1.34	79.21
Nagano	290,762	20,285	143.94	10.04	82.72
Gifu	230,072	272	118.23	0.14	72.30
Shizuoka	529,855	2551	147.92	0.71	102.98
Aichi	972,758	14,517	129.79	1.94	91.66
Mie	378,933	1111	217.53	0.64	97.24

**Table 8.2:** Number of claims, claims per 1000 population, and inpatient claims per inpatient (as of June 30) by prefecture, FY2022 (Shiga‐Okinawa).

Prefecture	Number of claims (inpatient)	Number of claims (outpatient)	Inpatient claims per 1000 population	Outpatient claims per 1000 population	Inpatient claims per inpatient (as of June 30)
Shiga	123,648	4772	87.76	3.39	67.94
Kyoto	177,563	2670	69.63	1.05	41.33
Osaka	923,180	15,479	105.12	1.76	61.62
Hyogo	894,657	4903	165.62	0.91	98.56
Nara	219,419	623	168.01	0.48	93.45
Wakayama	94,141	1956	104.25	2.17	63.61
Tottori	163,253	3177	300.10	5.84	121.56
Shimane	183,208	4145	278.43	6.30	100.39
Okayama	513,542	4502	275.80	2.42	137.31
Hiroshima	911,816	1744	330.37	0.63	122.42
Yamaguchi	525,872	6197	400.51	4.72	102.93
Tokushima	402,912	1218	572.32	1.73	132.49
Kagawa	285,801	3978	306.00	4.26	98.48
Ehime	311,752	3805	238.71	2.91	92.62
Kochi	304,488	2769	450.43	4.10	105.87
Fukuoka	1,980,374	18,597	387.09	3.64	119.99
Saga	519,273	863	648.28	1.08	144.44
Nagasaki	503,503	1861	392.44	1.45	80.75
Kumamoto	744,011	5586	433.07	3.25	101.39
Oita	439,286	0	396.83	0.00	95.98
Miyazaki	399,541	690	379.79	0.66	80.62
Kagoshima	726,725	6879	464.96	4.40	91.24
Okinawa	406,844	3058	277.14	2.08	93.96

## DISCUSSION

This study used NDB Open Data to analyze trends in the number of psychiatric OT claims in Japan, while acknowledging the dataset's structural limitations such as masking, lack of patient‐level information, and exclusion of paper‐based claims.

### Claims for inpatients

During the study period, the number of claims for inpatient psychiatric OT significantly increased. This increase appears to be attributable to both policy changes and expanding clinical needs. On the policy front, the 2018 revision of medical fees facilitated claims for psychiatric OT in dementia care wards, likely encouraging a broader implementation. From a clinical standpoint, despite a reported decrease in the number of inpatients according to the 630 Survey, the number of claims increased, suggesting both a growing demand and the potential for more intensive inpatient provision. An analysis by age group revealed distinct patterns. The highest absolute number of claims was observed among the elderly, particularly those aged ≥ 70 years, indicating that increases in these age groups strongly influenced the national trend. Analysis using claims per 1000 population and claims per inpatient (as of June 30) revealed that increases in some age groups could not be explained solely by demographic shifts.

For individuals aged ≥ 75 years, all metrics—claims, claims per 1000 population, claims per inpatient (as of June 30), and number of inpatients—increased. Projections using the NDB indicate that inpatient numbers will continue to rise through 2029,^24^ with long‐term inpatient numbers for this age group peaking in 2035.^25^ According to the 630 Survey, dementia was the most common diagnosis in this group. Policy changes in 2018, which allowed claims for psychiatric OT in dementia units, may have contributed to this increase. Patients admitted to psychiatric hospitals often present with severe behavioral and psychological symptoms of dementia (BPSD),^26^ accompanied by behavioral, functional, and physical issues, leading to a delayed discharge. These findings suggest the importance of initiating rehabilitation, including psychiatric OT, early during hospitalization. However, evidence regarding psychiatric OT for inpatients with dementia in Japan remains limited, necessitating further methodological development.

For those aged 65–74 years, although the number of inpatients decreased, the number of claims, claims per 1000 population, and claims per inpatient (as of the end of June) all increased significantly, indicating enhanced provision. According to the 2022 630 Survey, schizophrenia (F2) was the most common diagnosis (61.8%), followed by dementia (F0, 17.4%). This age group includes many long‐term inpatients, and interventions aimed at community reintegration and functional recovery, along with dementia care, were thought to contribute to the increase in the number of claims.

For individuals aged 40–64 and 20–39 years, while the number of inpatients and total claims decreased, claims per inpatient (as of June 30) increased slightly for those aged 20–39 years and significantly for those aged 40–64 years. This suggests a more intensive provision per patient, potentially reflecting strategies for discharge preparation and community living.

Under 20: Although the proportion of total claims was minimal, all indicators showed an increase. Children and adolescents encounter challenges such as parental emotional issues, academic stress, and poverty.^27^ Suicide continues to be a leading cause of mortality,^28^ with 40%–80% of adolescent psychiatric patients reporting self‐harm.^29^ Research on psychiatric OT in suicide prevention is limited^30^; however, occupational therapists, as specialists in addressing work that fosters purpose and values—potential factors in suicide prevention—may play a role in prevention. In 2022, developmental disorders (F8) emerged as the most prevalent diagnosis (24.5%), surpassing schizophrenia (F2, 15.2%). The characteristics of developmental disorders, such as reduced stress tolerance, communication difficulties, and rigid thinking, can hinder participation in educational and societal contexts. Given that most Japanese psychiatric OT research focuses on schizophrenia, further development and dissemination of approaches for developmental disorders and other conditions are necessary. Limitations include the fact that the 630 Survey reflects the number of inpatients at a single point in time (June 30) rather than the annual total, and that the NDB public data lack detailed information such as length of stay, diagnosis names, and medical history. Future research should utilize patient data from 2023 onwards and integrate multiple datasets to comprehensively understand psychiatric OT provision in inpatient settings.

### Claims for outpatients

The results showed that the number of psychiatric OT claims for outpatient decreased after the COVID‐19 pandemic, and the number of claims by age group also showed a significant decrease in all age groups before and after the COVID‐19 pandemic. Psychiatric daycare, a component of outpatient rehabilitation, exhibited a declining trend both prior to and following the COVID‐19 pandemic.^16^ A similar pattern was observed in psychiatric OT. In countries outside Japan, the lockdowns implemented since the fiscal year 2020 have been reported to decrease the number of visits to outpatient mental health services and outpatient rehabilitation, including psychiatric daycare.^31^, ^32^ Many facilities considered reducing or suspending outpatient psychiatric OT due to infection control measures, potentially contributing to a decline in service utilization. The observed reduction in claims for psychiatric OT, both before and after the pandemic, cannot be solely attributed to a decrease in outpatient visits. According to the “Patient Survey”^33^ conducted by the Ministry of Health, Labor and Welfare, while adjustments for changes in survey methodology are necessary, there is an upward trend in the number of patients. The decline in claims, despite an increasing patient population, indicates that factors such as infection control measures, including restrictions on group programs and staff shortages, may have rendered the provision of OT physically challenging. In Japan, the existing reimbursement framework for psychiatric OT stipulates two‐hour group‐based sessions. This duration requirement may impede access for patients who struggle to engage in activities for prolonged periods, thereby restricting the flexibility to offer shorter or individualized interventions that may more effectively address their needs. Future reimbursement reforms should consider including more adaptable formats, such as shorter duration and individualized sessions, to enhance the inclusivity and responsiveness of psychiatric OT. Furthermore, unlike psychiatric daycare facilities, which provide a structured framework for time and space, psychiatric OT is program‐based, facilitating the implementation of patient‐centered interventions, such as occupation‐based practices. The highest number of outpatient visits was recorded for individuals in their 40s. This demographic particularly requires interventions aimed at promoting independent living and social reintegration within the community, as their aging parents will soon be unable to provide ongoing support, necessitating their independence in the near future. Therefore, psychiatric OT is a valuable approach for supporting outpatients and should be offered to a broad patient population. Consequently, it is crucial to monitor trends in outpatient visits as the COVID‐19 pandemic subsides. Continuity of care through coordinated inpatient‐to‐outpatient rehabilitation pathways should be strengthened.

### Sex difference

As a result of the analysis of sex differences in the number of psychiatric OT claims for inpatients, it was confirmed that the number of claims was significantly higher among females. In particular, in the age group of ≥75 years, where the number of calculations was the highest, the number of female inpatients was approximately 1.8 times that of male inpatients (630 Survey: 2022). This suggests that the high number of female inpatients in the relevant age group may have contributed to the sex difference in the number of times performed. In addition, it is important to reconsider the content of occupational activities that are more acceptable and compatible with women in this age group to realize effective interventions.

Conversely, no significant difference was observed in the number of claims for outpatients between males and females. This finding contrasts with previous studies that have identified sex differences in psychiatric daycare,^16^ which is also administered on an outpatient basis. Psychiatric daycare is characterized by extended session durations and its role as a “place to be” for its participants. In contrast, psychiatric OT is distinctly characterized as a single treatment program completed within a relatively short time frame. Due to these differences in implementation, psychiatric OT is less likely to exhibit gender differences in participation ease, which may explain the absence of gender differences in the frequency of participation.

### Regional difference

The findings indicate substantial regional disparities in psychiatric OT implementation, with the most pronounced differences exceeding the average by several times. Inpatient psychiatric OT showed variations of 21.0 times in claims, 9.3 times in claims per population, and 3.5 times in claim rate per inpatient. A positive correlation existed between psychiatric beds per population and both claims per population and claim rate per inpatient. This suggests regions with more beds may have better‐equipped staff and facilities, enabling psychiatric OT integration into treatment. However, this interpretation needs caution. In Japan, psychiatric bed overprovision and extended hospital stays remain ongoing concerns. More beds do not necessarily lead to earlier discharge or community transition. Consequently, psychiatric OT should be systematically implemented with explicit objectives, such as promoting discharge and facilitating community transitions. Moving forward, it is crucial to integrate data on intervention objectives, program content, and staff allocation to identify factors contributing to regional differences and establish appropriate provision systems.

### Implication

The results of this study provide important implications for both clinical practice and mental health policy. Clinically, the increased use of inpatient psychiatric OT among elderly and younger populations highlights the need for age‐specific therapeutic approaches. For elderly patients, interventions that support cognitive functioning, maintain daily living skills, and promote engagement in familiar routines are essential, particularly in long‐term psychiatric wards. Among younger patients, including adolescents and young adults, it is increasingly important to implement programs that address emotional regulation, social functioning, and particularly self‐harm or suicide prevention—an area of growing concern in recent years.

The marked decline in outpatient psychiatric OT, particularly after the onset of the COVID‐19 pandemic, emphasizes the fragility of community‐based rehabilitation systems under public health crises. Maintaining the continuity of rehabilitation services during pandemics or other emergencies should be a fundamental component of psychiatric service planning. To support this goal, it is essential to establish systems that enable the timely sharing of local innovations and practical strategies across facilities. Professional organizations may play a key role in facilitating such platforms, allowing frontline staff to learn from each other's adaptations and promote continuity in therapeutic practice. In the future, systems that enable the timely exchange of practical knowledge across facilities—such as platforms for sharing brief reports or case‐based insights—may help promote adaptive and coordinated responses in times of crisis. While this study did not directly assess facility‐level responses, the observed nationwide decline in outpatient services highlights the vulnerability of psychiatric rehabilitation to external disruptions. These findings may underscore the potential value of enhancing communication channels among practitioners to support adaptive service delivery, especially during times of crisis.

From a policy perspective, the sharp decrease in outpatient service delivery raises concerns about barriers faced by people with mental illness who are striving to live independently and meaningfully in the community. Psychiatric OT plays a vital role in supporting self‐determination, recovery, and community participation, particularly among the working‐age population and those living outside institutional settings. Policymakers should consider revising medical reimbursement schemes to support sustained outpatient OT provision, strengthening staffing systems, and ensuring that OT is integrated as a core component of inclusive and person‐centered mental health care systems. In particular, reforming the current reimbursement structure—traditionally focused on two‐hour group interventions—by introducing more flexible billing options, such as short‐duration individual sessions similar to physical rehabilitation, may enable broader access and responsiveness to individual needs. Such measures are essential to meet evolving demographic demands and to reinforce the resilience of psychiatric rehabilitation systems in future health crises.

### Conclusion

This study examined the current status and trends in psychiatric OT claims using NDB Open Data and investigated the factors influencing these changes. The findings revealed an increase in the total number of claims for inpatients, with a particularly notable trend among elderly patients and those aged < 20 years. This trend is likely attributable to an increase in the number of elderly inpatients with dementia and a growing prevalence of mental health issues among younger individuals. These developments underscore the increasing importance of psychiatric OT during hospitalization. Conversely, the number of claims for outpatient has declined since the onset of the coronavirus disease (COVID‐19) pandemic. This decline may be attributed to the impact of scale reduction and discontinuation of operations associated with infection control measures. However, psychiatric OT is anticipated to be actively utilized as a supportive option for outpatients by leveraging its unique characteristics. The results of this study offer insights into future psychiatric OT practices and policy development. Specifically, enhancing support for the elderly, whose numbers are expected to rise, expanding the application of psychiatric OT for younger populations, and promoting the utilization of outpatient rehabilitation are critical challenges in this field. To address these issues, further evidence on specific intervention methods for psychiatric OT is required. This study had several limitations. The first limitation is that the NDB Open Data do not include patient‐specific information such as diagnoses, medical history, or treatment. To address this limitation, we also referred to the results of the 630 Survey; however, the information on patients diagnosed with psychiatric OT was not directly reflected. Second, while the NDB Open Data offer high comprehensiveness, they exclude paper‐based claims. This may result in underrepresentation of facilities that continued using paper submissions during the study period, such as smaller institutions or psychiatric hospitals. Furthermore, data from claim forms for medical expenses covered solely by public funds were not included. Accordingly, the findings should be interpreted with caution, particularly when considering national representativeness. Third, this study exclusively examined psychiatric OT interventions billed under the specific reimbursement code for psychiatric OT in the national fee schedule. OT services provided in psychiatric settings without separate billing, such as those included in certain bundled payment systems (e.g., child/adolescent psychiatric inpatient care fee), were not captured in the NDB dataset. Therefore, the results may underestimate the overall provision of OT in psychiatric care.

## AUTHOR CONTRIBUTIONS

Tomoyuki Okazaki conceived and designed the study, performed the data analysis, and drafted the manuscript. Satoshi Asaoka supervised the study, verified the reproducibility of the analyses, and contributed to the interpretation of results. Chie Kitano contributed to the development and refinement of the discussion section, focusing on clinical relevance. Hitoshi Okamura provided overall supervision, advised on analytical strategy and presentation, and critically reviewed the manuscript.

## CONFLICT OF INTEREST STATEMENT

The authors declare no conflicts of interest.

## ETHICS APPROVAL STATEMENT

N/A.

## PATIENT CONSENT STATEMENT

N/A.

## CLINICAL TRIAL REGISTRATION

N/A.

## Data Availability

The data that support the findings of this study are available in NDB Open Data at https://www.mhlw.go.jp/stf/seisakunitsuite/bunya/0000177182.html. The data that support the findings of this study are available in the 630 Survey at https://www.ncnp.go.jp/nimh/seisaku/data/630.html. The data that support the findings of this study are available in the population estimates provided by the Ministry of Internal Affairs and Communications at https://www.e-stat.go.jp/statsearch/files?page=1&layout=datalist&toukei=00200524&tstat=000000090001&cycle=7&tclass1=000001011679&tclass2val=0. The data that support the findings of this study are available in the Survey of medical institutions (2022) at https://www.e-stat.go.jp/stat-search/files. These data were derived from the following resources available in the public domain: NDB Open Data, https://www.mhlw.go.jp/stf/seisakunitsuite/bunya/0000177182.html; 630 Survey, https://www.ncnp.go.jp/nimh/seisaku/data/630.html; The population estimate, https://www.e-stat.go.jp/statsearch/files?page=1&layout=datalist&toukei=00200524&tstat=000000090001&cycle=7&tclass1=000001011679&tclass2val=0; and Survey of medical institutions (2022), https://www.e-stat.go.jp/stat-search/files. Public data (NDB, hospital bed counts, population statistics, 630 Survey) were used and are available upon request.

## References

[1] World Health Organization . https://www.who.int/news-room/fact-sheets/detail/mental-disorders. Accessed 27 Mar 2025.

[2] Ministry of Health, Labour and Welfare of Japan. https://www.mhlw.go.jp/content/11121000/001374464.pdf. Accessed 27 Mar 2025.

[3] OECD Reviews of Health Care Quality JAPAN. https://www.oecd.org/content/dam/oecd/en/publications/reports/2015/08/oecd-reviews-of-health-care-quality-japan-2015_g1g4c40f/9789264225817-en.pdf. Accessed 3 Apr 2025.

[4] WFOT. https://wfot.org/about/about-occupational-therapy. Accessed 8 Sep 2025.

[5] Shinozaki A , Hayashi T , Okamura H . Effects of a psychoeducation program for people with schizophrenia aimed at increasing subjective well‐being and the factors influencing those effects: a preliminary study. Psychiatr Q. 2020 Mar;91:45–52. 31768909 10.1007/s11126-019-09679-4

[6] Shimada T , Ohori M , Inagaki Y , Shimooka Y , Sugimura N , Ishihara I , et al. A multicenter, randomized controlled trial of individualized occupational therapy for patients with schizophrenia in Japan. PLoS One. 2018 Apr 5;13(4):e0193869. 29621261 10.1371/journal.pone.0193869 PMC5886394

[7] Rocamora‐ Montenegro M , Compañ‐ Gabucio L‐M , Garcia de la Hera M . Occupational therapy interventions for adults with severe mental illness: a scoping review. BMJ Open. 2021 Oct 29;11(10):e047467. 10.1136/bmjopen-2020-047467 PMC8559113 34716157

[8] Tatsumi E , Yotsumoto K , Nakamae T , Hashimoto T . Effects of occupational therapy on hospitalized chronic schizophrenia patients with severe negative symptoms. Kobe J Med Sci. 2012 May 24;57(4):E145-54. 22971985

[9] Tanaka C , Yotsumoto K , Tatsumi E , Sasada T , Taira M , Tanaka K , et al. Improvement of functional independence of patients with acute schizophrenia through early occupational therapy: a pilot quasi‐experimental controlled study. Clin Rehabil. 2014 Aug;28:740–747. 24554687 10.1177/0269215514521440

[10] Thomas EC , Read H , Neumann N , Zagorac S , Taylor C , Kramer I , et al. Implementation of occupational therapy within early intervention in psychosis services: results from a national survey. Early Interv Psychiatry. 2023 Jul;17:652–661. 36192371 10.1111/eip.13359

[11] Ministry of Health, Labor and Welfare Insurance, Medical Economics Division. Summary of the 2018 revision of medical fees. https://www.mhlw.go.jp/file/06-Seisakujouhou-12400000-Hokenkyoku/0000198532.pdf. Accessed 27 Mar 2025.

[12] Definition of Occupational Therapy in Japan. https://wfot.org/member-organisations/japan-japanese-association-of-occupational-therapists. Accessed 23 Feb 2025.

[13] Japanese Association of Occupational Therapist. White paper on occupational therapy‐2021 (in Japanese). https://www.jaot.or.jp/files/page/jimukyoku/OT_whitepaper2021.pdf. Accessed 27 Mar 2025.

[14] Murai C , Endo C . Ima Seishinka no Sagyoryohoshi ga dekirukoto [What psychiatric occupational therapists can do today] (in Japanese). J Jpn Psychiatr Hosp Assoc. 2023;42(3):97–102.

[15] Higuchi S , Sako A , Kondo T , Kusanishi S , Enomoto T , Hayakawa T , et al. Clozapine use in Japan based on National Database of Health Insurance Claims and specific health checkups open data: disparities by region, age, and sex (in Japanese). Psychiatr Neurol Jpn. 2022;124:3–15. Available from: https://journal.jspn.or.jp/jspn/openpdf/1240010003.pdf

[16] Okazaki T , Asaoka S , Okamura H . Trends in psychiatric day care practices in Japan: an analysis using the National Database of Health Insurance Claims open data. Cureus. 2024 Jun 25;16(6):e63166. 10.7759/cureus.63166 39070311 PMC11272950

[17] Hori S , Ushida K , Momosaki R . Trends in outpatient rehabilitation practices in Japan: analysis using the National Database of Health Insurance Claims Open Data. J Rural Med. 2022;17:125–130. 10.2185/jrm.2021-051 35847760 PMC9263955

[18] Uchino S , Taguri M . Epidemiology of cruciate ligament surgery in Japan: a repeated cross‐sectional study from 2014 to 2021. PLoS One. 2023 Dec 22;18(12):e0288854. 10.1371/journal.pone.0288854 38134038 PMC10745212

[19] Ministry of Health, Labour and Welfare, Health Insurance Bureau, Division for Health Care and Long‐term Care Integration. The 9th time NDB Open Data comment volume (in Japanese). Accessed 23 Feb 2025.

[20] National Institute of Mental Health, National Center of Neurology and Psychiatry, Japan. Annual data of mental health in Japan. https://www.ncnp.go.jp/nimh/seisaku/data/630.html. Accessed 27 Mar 2025.

[21] Ministry of Internal Affairs and Communications: Population Estimates. https://www.e-stat.go.jp/statsearch/files?page=1&layout=datalist&toukei=00200524&tstat=000000090001&cycle=7&tclass1=000001011679&tclass2val=0. Accessed 8 Sep 2025.

[22] Survey of medical institutions. https://www.e-stat.go.jp/stat-search/files (2022). Accessed 28 Aug 2025.

[23] Shimizu H . An introduction to the statistical free software HAD: suggestions to improve teaching, learning and practice data analysis (in Japanese). J Media Inf Commun. 2022;1:59–73.

[24] Tadashi T , Toshiaki K , Kentaro U , Yasuyuki O , Masato F , Koji Y , et al. Changes in psychiatric inpatient care from a statistical perspective (in Japanese). Psychiatr Neurol Jpn. 2023;125:762–770. 10.57369/pnj.23-108

[25] Okayama T , Usuda K , Okazaki E , Yamanouchi Y . Number of long‐term inpatients in Japanese psychiatric care beds: trend analysis from the patient survey and the 630 Survey. BMC Psychiatry. 2020 Nov 3;20(1):522. 10.1186/s12888-020-02927-z 33143670 PMC7607734

[26] Yoshimura A , Lebowitz A , Bun S , Aiba M , Ikejima C , Asada T . A comparative analysis of dementia inpatient characteristics: results from a nationwide survey of different care facilities in Japan. Psychogeriatrics. 2016 Jan;16(1):34–45. 10.1111/psyg.12117 25919913

[27] Armitage JM , Collishaw S , Sellers R . Explaining long‐term trends in adolescent emotional problems: what we know from population‐based studies. Discov Soc Sci Health. 2024;4:14. 10.1007/s44155-024-00076-2

[28] World Health Organization—Suicide. https://www.who.int/news-room/fact-sheets/detail/suicide. Accessed 27 Mar 2025.

[29] Klonsky ED , Muehlenkamp JJ . Self‐injury: a research review for the practitioner. J Clin Psychol. 2007 Nov;63(11):1045–1056. 10.1002/jclp.20412 17932985

[30] Marshall CA , Crowley P , Carmichael D , Goldszmidt R , Aryobi S , Holmes J , et al. Effectiveness of suicide safety planning interventions: a systematic review informing occupational therapy. Can J Occup Ther. 2023 Jun;90(2):208–236. 10.1177/00084174221132097 36324257 PMC10189833

[31] Prina E , Tedeschi F , Salazzari D , Botte T , Ballarin M , Rabbi L , et al. Effect of COVID‐19 pandemic on utilization of community‐based mental health care in North‐East of Italy: a psychiatric case register study. Epidemiol Psychiatri Sci. 2023 Apr 11;32:e17. 10.1017/S2045796023000100 PMC10130733 37039429

[32] Ryu S , Nam HJ , Baek SH , Jhon M , Kim JM , Kim SW . Decline in hospital visits by patients with schizophrenia early in the COVID‐19 outbreak in Korea. Clin Psychopharmacol Neurosci. 2022;20:185–189. 35078961 10.9758/cpn.2022.20.1.185 PMC8813312

[33] Patient Survey. https://www.mhlw.go.jp/toukei/list/10-20.html. Accessed 8 Sep 2025.

